# Correlation of 18F-FDG PET/MRE Metrics with Inflammatory Biomarkers in Patients with Crohn's Disease: A Pilot Study

**DOI:** 10.1155/2017/7167292

**Published:** 2017-09-19

**Authors:** Liran Domachevsky, Haim Leibovitzh, Irit Avni-Biron, Lev Lichtenstein, Natalia Goldberg, Meital Nidam, David Groshar, Hanna Bernstine, Ofer Ben-Bassat

**Affiliations:** ^1^Department of Nuclear Medicine, Assuta Medical Centers and Sackler Faculty of Medicine, Tel Aviv University, Tel Aviv, Israel; ^2^IBD Center, Division of Gastroenterology, Rabin Medical Center, Petah Tikva, Israel

## Abstract

**Background:**

To investigate the association between 18F-FDG (Fluorodeoxyglucose) PET (positron emission tomography)/MRE (magnetic resonance enterography) metrics with the inflammatory biomarkers fecal calprotectin and C-reactive protein (CRP) in patients with Crohn's disease (CD).

**Methods:**

This prospective pilot study was institutional review board (IRB) approved with informed consent obtained. Consecutive CD patients were referred to 18F-FDG PET/MRE. Patients in whom colonoscopy was performed and CRP and fecal calprotectin levels were measured were included. CRP and fecal calprotectin were regarded as positive for inflammation if they were greater than 0.5 mg/dl and 150 mcg/g, respectively. Correlation of quantitative variables was performed using the Pearson's correlation coefficient. Receiver operating characteristic (ROC) curves were drawn and the area under the curve (AUC) was calculated to evaluate the accuracy of PET and MRE metrics in determining the presence of inflammation evaluated by calprotectin and CRP levels.

**Results:**

Analysis of 21 patients (16 women and 5 men, 43 ± 18 years) was performed. Magnetic resonance index of activity (MaRIA) score had an AUC of 0.63 associated with fecal calprotectin and CRP. Adding apparent diffusion coefficient (ADC) and metabolic inflammatory volume (MIV) to MaRIA score resulted in an AUC of 0.92 with a cutoff value of 447 resulting in 83% and 100% sensitivity and specificity, respectively.

**Conclusion:**

The addition of ADC and MIV to the MaRIA score increases the accuracy for discrimination of disease activity in patients with CD.* Trial registration number* is 2015062.

## 1. Introduction

Crohn's disease (CD) is a chronic, relapsing, transmural inflammatory disease that can affect the entire gastrointestinal track [1]. Diagnosis and treatment assessment are based on clinical, endoscopic and cross-sectional imaging. However, clinical symptoms and clinical scores do not always correlate with endoscopic findings [2] and have not changed the long-term outcome in patients with CD [3]. Endoscopy on the other hand is very accurate in the assessment of early manifestations and enables histological evaluation of inflammation; however, it is an invasive tool that assesses the mucosal layer and only a short segment of the small bowel (i.e., terminal ileum).

The introduction of “treat to target” paradigm in CD with mucosal healing defined as the target, has led to search for convenient, reliable, and quantifiable variables to predict and assess the response to therapy and to monitor CD patients [3]. At present, C-reactive protein (CRP) and fecal calprotectin are the most widely used inflammatory biomarkers as a surrogate to endoscopy to monitor patients with CD [4]. Nevertheless, both tests have limitations and more objective tools are required.

Computerized tomography enterography (CTE) and Magnetic resonance enterography (MRE) are complementary diagnostic tools in the work-up of patients with CD as both can identify pathological processes in deeper layers of the bowel and extraintestinal findings and evaluate the entire GI tract.

MRE has gained popularity given the lack of ionizing radiation [5], high contrast resolution, and the ability to perform dynamic contrast imaging. It was shown to have similar diagnostic accuracy as compared to CTE [6]. The addition of more advanced sequences such as diffusion weighted imaging (DWI) has increased the diagnostic accuracy [6]. Magnetic resonance index of activity (MaRIA) is MRE-based score that has been found to be reliable in quantifying the severity of the inflammatory processes in patients with CD and in assessing the response to therapy [7].

18-Fluorodeoxyglucose (18F-FDG) uptake is seen in metabolically active cells including inflammatory cells. Increased 18F-FDG uptake on PET/CTE was proven to be sensitive (54–98%) and specific (55–81%) in identifying active inflammatory segments of the small and large bowel in patients with CD. Uptake can be assessed qualitatively and semiquantitatively and therefore can be used to evaluate objectively the degree of inflammation and response to treatment [8]. However, given the high patient radiation dose, the study has not gained popularity, particularly as most patients with CD are young and repeated studies are necessary.

Simultaneous 18F-FDG PET/MRE might combine the advantages of both modalities. Several studies have recently demonstrated the feasibility of PET/MRE [9] and have shown the superiority of PET/MRE compared to PET/CT and MRE in the detection of extraluminal disease and in differentiating fibrotic from inflammatory components [10]. The aim of this prospective pilot study is to evaluate the association between 18F-FDG PET/MRE metrics with the inflammatory biomarkers fecal calprotectin and CRP.

## 2. Materials and Methods

This prospective study has been approved by the institutional review board. All subjects signed an informed consent form. Between December 2015 and December 2016, consecutive patients with newly diagnosed CD or patients with a known CD presenting with a flare-up were referred to 18F-FDG PET/MRE. All patients were off corticosteroid or biologic treatment. Patients were included in the study only if colonoscopy and laboratory work-up have been performed prior to or after 18F-FDG PET/MRE within 6 and 2 weeks, respectively. Laboratory work-up included fecal calprotectin, blood C-reactive protein (CRP) levels, white blood cell counts (WBC), platelets count, hemoglobin, hematocrit, creatinine, and albumin levels. CRP and fecal calprotectin were regarded as positive for inflammation if greater than 0.5 mg/dl and 150 mcg/g, respectively.

### 2.1. 18 F FDG PET/MRE Protocol

Patients were required to fast at least for 4 hours prior to arrival to the department. Upon arrival an intravenous catheter was placed for radiopharmaceutical administration, and for glucose level measurement. Patients received an intravenous injection of 3-4 mCi of 18F FDG (estimated effective dose of 1.87–2.5 mSv [11]). During the 18F FDG uptake phase of 45 minutes, patients were asked to drink a total volume of 2 ml/kg Avilac syrup 66.7 g/100 ml–300 ml (Perrigo, IL) diluted in 1000 ml of water (i.e., a total of 1050–1200 ml, 175–200 ml every 7 minutes for 42 minutes) for optimal small bowel distention and to achieve high contrast resolution between bowel wall and lumen on T2 weighted and postcontrast imaging.

18F-FDG PET/MR was performed from the diaphragm to the mid-thigh on the Biograph mMR (Siemens AG, healthcare sector, Erlangen, Germany) simultaneous PET/MR system. Patients were positioned supine and multistep/multibed scanning was performed in caudocranial direction with two bed positions. We used a 24-channel spine RF coil integrated within the MR bed and 2 surface body coils (6 channel each) to cover the abdomen and pelvis. MR sequences included coronal thick slab T2-weighted image to determine that oral contrast has reached the right colon followed by intramuscular injection of 1 mg Glucagon is administered. Coronal and axial (with and without fat suppression) T2-weighted half-Fourier acquisition single shot turbo spin echo (HASTE) image, axial T1-weighted volumetric interpolated breath-hold examination (VIBE) (with and without fat suppression), DWI (*b* = 50, 500, 1000, and 1600 sec/mm^2^), coronal T1-weighted nonenhanced VIBE image and gadolinium enhanced coronal T1-weighted VIBE image with 30 and 70 seconds delays, and axial T1-weighted VIBE image with 95 seconds delay. We used Gadoteric acid (Dotarem®, Guerbet, France) (0.2 ml/kg, 0.1 mmol/kg at 1-2 ml/s, 20 ml saline flush) as intravenous contrast media.

PET data was acquired in the list mode with the following reconstruction parameters: High definition PET + ordered subset expectation maximization (OSEM) iterative algorithm, three iteration and 21 subsets, Gaussian filter: FWHM 4 mm; relative scattered correction.

### 2.2. Image Analysis

#### 2.2.1. PET Metrics

**Figure 1:**
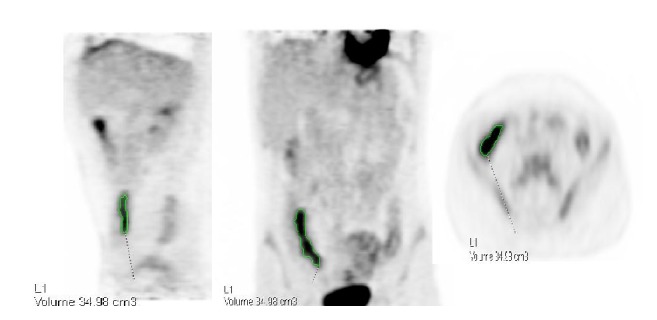
Metabolic inflammatory volume (MIV) calculation using isocontour application with spheres drawn over bowel segments with increased FDG uptake using a fixed 40% threshold.

We used dedicated software for PET metric measurements (Syngo.via; Siemens AG, healthcare sector, Erlangen, Germany). A sphere VOI was drawn on bowel segments with pathological 18F FDG uptake (i.e., above physiological 18F FDG uptake in normal appearing bowel segments) and SUVmax was calculated. The metabolic inflammatory volume (MIV) was meticulously calculated using isocontour application with spheres drawn over bowel segments with increased FDG uptake using a fixed 40% threshold (Figure 1). To avoid false positive FDG uptake due to physiologic activity all segments were compared with MR images to confirm abnormal appearance on MR. All spherical VOI were visually evaluated on axial, sagittal, and coronal planes to be certain that the VOI is well located. In addition, the length of visually pathological FDG-avid segments was measured.

Normalization for body weight was performed using the patient weight in kg, measured before 18F FDG injection.

#### 2.2.2. MRI Metrics

The following variables were evaluated [1]: Bowel wall thickness measured in mm, edema (i.e., high signal on T2-weighted imaged with fat suppression), bowel wall enhancement (i.e., higher than the enhancement of normal appearing segment), mesenteric vascularity (comb sign) and edema, the presence of enlarged mesenteric lymph nodes, the presence of ulcer/fistula/fibrosis or abscess, and the length of the visually abnormal appearing segments measured on MRI and PET images. In case of skip lesions the sum of lengths was calculated.

DWI/ADC: region of interest (ROI) was placed over different locations in the affected segments with the highest signal intensity on DWI. The minimal ADC value was recorded.

MaRIA score [12]: 1.5 × wall thickness (mm) + 0.02 × relative contrast enhancement (RCE) + 5 × edema + 10 × ulceration. RCE = (wall signal intensity (WSI) after gadolinium − WSI before gadolinium)/(WSI before gadolinium) × 100 × SD noise before gadolinium/SD noise after gadolinium.

All measurements were conducted in consensus by a dual board-certified in radiology and nuclear medicine physician (LD, with 6 years of experience) and a board-certified nuclear medicine physician (HB, with 10 years of PET/CT experience).

### 2.3. Statistical Analysis

Correlation of quantitative variables was performed using the Pearson's correlation coefficient.

Receiver operating characteristic (ROC) curves were drawn and the area under the curve (AUC) was calculated to evaluate the accuracy of different PET and MRE metrics in determining the presence or lack of inflammation evaluated by calprotectin and CRP levels either separately or combined.

*p* ≤ 0.05 was considered statistically significant. All data was analyzed using Medcalc (version 17.5.5, 2017).

## 3. Results

Twenty-seven consecutive patients (20 women and 7 men, 42 ± 15 years) were prospectively recruited to the study. All patients underwent 18F-FDG PET/MRE. Analysis was performed on 21 patients (16 women and 5 men, 43 ± 18 years) (three patients had no calprotectin levels available and three refused to undergo colonoscopy).

**Figure 2:**
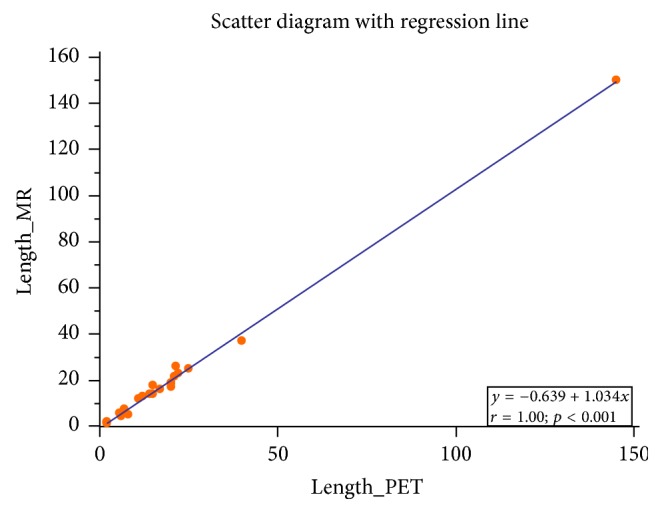
Scatter diagram with regression line demonstrating very high correlation between the length of the involved segments measured on PET and MR images.

**Figure 3:**
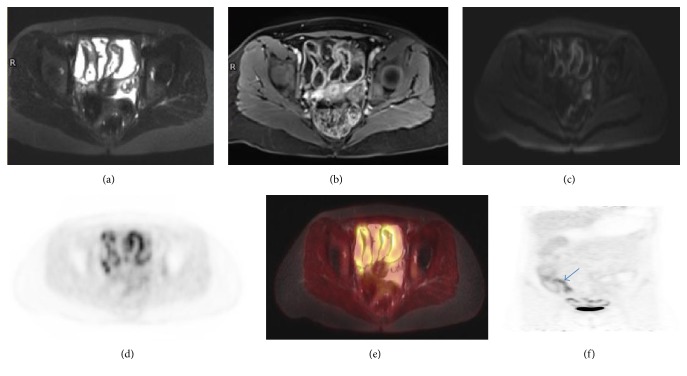
29-year-old woman with CD. (a) Axial T2-weighted FS image demonstrating thickened wall of a long small bowel segment. (b), (c) There is increased enhancement and restricted diffusion of the involved small bowel mucosa seen on axial contrast enhanced T1-weighted and DWI images, respectively. (d) Axial PET attenuation correction image demonstrating FDG uptake along the involved small bowel segments. (e) Axial fused T2-weighted FS PET/MR image demonstrating FDG uptake correlating with thickened small bowel wall. (f) Coronal PET attenuation correction image showing increased FDG uptake in the terminal ileum (arrow) and several small bowel segments. Colonoscopy revealed terminal ileitis that was further confirmed by histology. However, colonoscopy did not reflect the true extent of disease as seen on 18F-FDG PET/MR.

The length of the involved segments on PET attenuated corrected (AC) images correlated with the length as measured on MRI with* R*^2^ of 0.99 (Figures 2 and 3).

**Figure 4:**
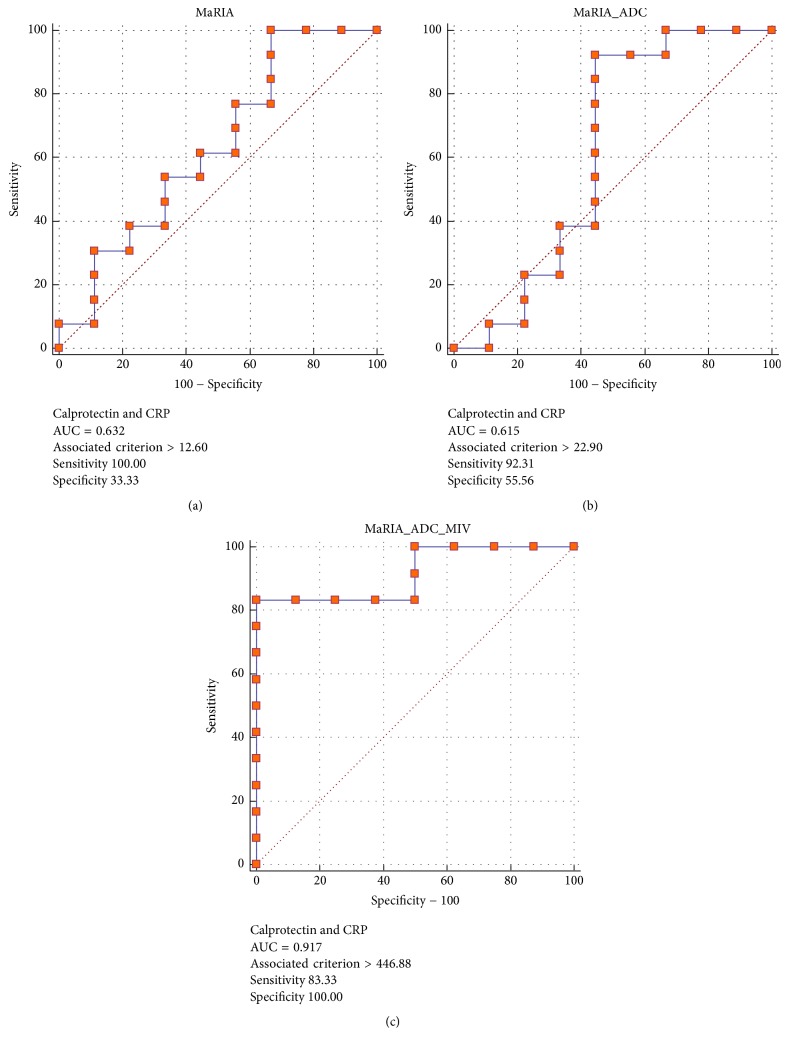
Receiver operating characteristic curves using calprotectin and CRP levels to determine active versus nonactive disease with MaRIA (a), MaRIA and ADC (b), and MaRIA, ADC, and MIV (c) as the variables. CRP: C-reactive protein; MaRIA: magnetic resonance index of activity; ADC: apparent diffusion coefficient; MIV: metabolic inflammatory volume.

**Figure 5:**
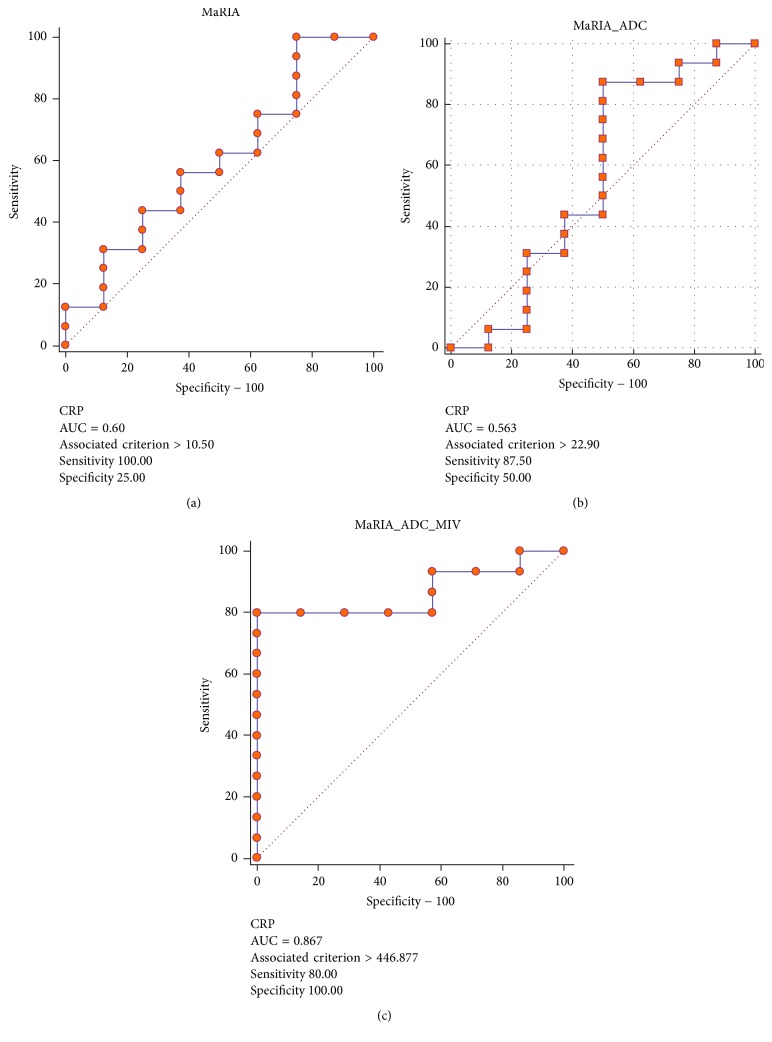
Receiver operating characteristic curves using CRP levels to determine active versus nonactive disease with MaRIA (a), MaRIA and ADC (b), and MaRIA, ADC, and MIV (c) as the variables. CRP: C-reactive protein; MaRIA: magnetic resonance index of activity; ADC: apparent diffusion coefficient; MIV: metabolic inflammatory volume.

**Figure 6:**
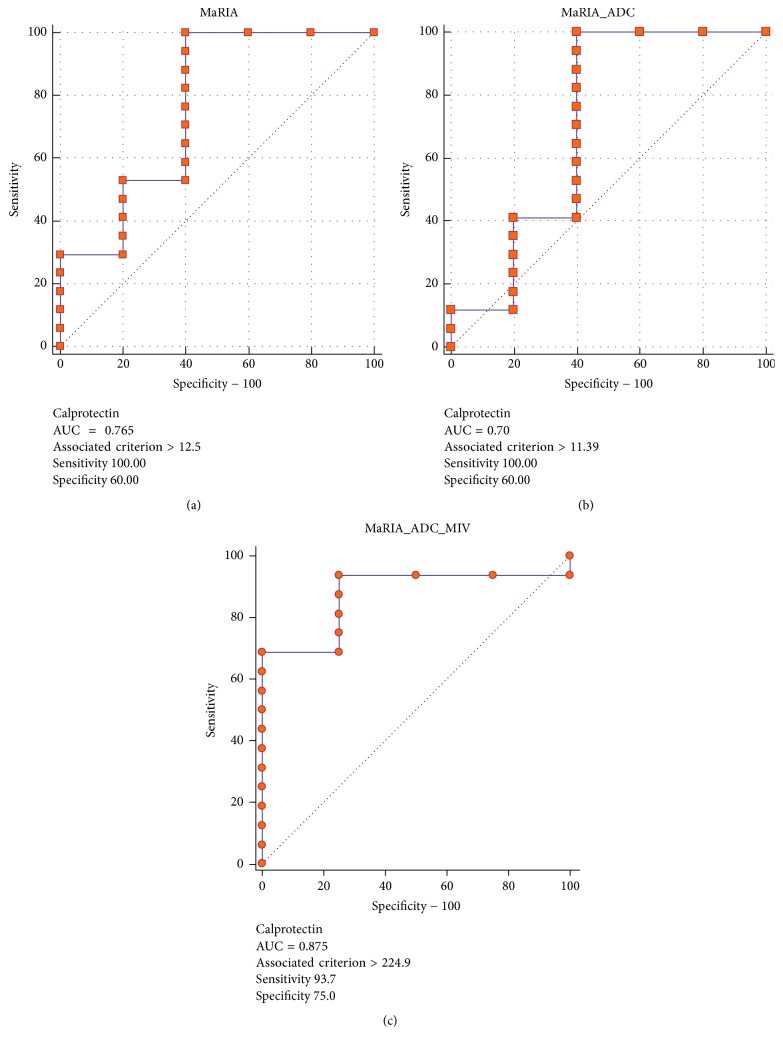
Receiver operating characteristic curves using calprotectin levels to determine active versus nonactive disease with MaRIA (a), MaRIA and ADC (b), and MaRIA, ADC, and MIV (c) as the variables. MaRIA: magnetic resonance index of activity; ADC: apparent diffusion coefficient. MIV: metabolic inflammatory volume.

MaRIA score had an AUC of 0.72 associated with fecal calprotectin. Adding ADC and MIV to MaRIA score (i.e., MaRIA × ADC × MIV/1000) resulted in an AUC of 0.88. MaRIA score had an AUC of 0.6 associated with CRP. Combining ADC and MIV to MaRIA score resulted in an AUC of 0.87. When fecal calprotectin and CRP were combined to differentiate active from nonactive disease (i.e., inflammation was determined only if both tests were abnormal), MaRIA score had an AUC of 0.63 associated with fecal calprotectin and CRP. Adding ADC and MIV to MaRIA score resulted in an AUC of 0.92 with a cutoff value of 447 resulting in 83% and 100% sensitivity and specificity, respectively (Figures 4–6). The AUC for SUVmax, SUVmax and MaRIA score and SUVmax, MaRIA score and MIV when fecal calprotectin and CRP were combined was 0.63, 0.6, and 0.8, respectively.

## 4. Discussion

The results of this pilot study imply that 18F-FDG PET/MRE metrics of MIV and ADC have an added value to MaRIA score in discriminating patients with active from nonactive Crohn's disease based on fecal calprotectin and CRP levels.

At present the diagnosis and treatment of patients with CD rely on clinical and laboratory evaluation, endoscopic assessment, and cross-sectional imaging [13]. However, the new concept of “treat to target” in CD patients, with mucosal healing gaining importance as the target aimed at, necessitates a noninvasive, reliable tool to monitor patients and to assess disease activity. Among several biomarkers that have been proposed to surrogate endoscopy in order to evaluate active inflammation and mucosal healing in CD, fecal calprotectin and CRP are currently the most widely used. Fecal calprotectin is released from human neutrophils and macrophages and reflects mucosal inflammation. It was found to be correlated with endoscopy in detecting disease activity and severity of intestinal inflammation. Abej et al. [14] have shown that stool calprotectin can differentiate active from nonactive disease and correlated with endoscopic findings. Calprotectin has been also used to monitor response to therapy and predict relapse [15]. Sipponen et al. [16] showed that normalization of calprotectin values corresponds to response to therapy on endoscopy while for patients with sustained abnormal values no improvement was seen on endoscopy.

CRP has long become the biomarker of choice for assessment of inflammatory CD activity and was found to be more reliable in cases of transmural inflammation [4]. It is produced and released from hepatocytes and its synthesis is stimulated by interleukin 6 [17]. The relatively short half-life of 19 hours makes it sensitive to early changes in the status of inflammation. CRP has been shown to correlate with clinical activity [18] and endoscopic findings [19] to predict clinical relapse [20] and to assess the response to therapy [21]. However both biomarkers have their pros and cons in determining the status of CD. For example, single nucleotide polymorphism in CRP genes is known to affect the baseline and stimulated CRP levels resulting in false negative results [22]. Additionally, CRP might be less sensitive to mucosal inflammation and hence underestimate subtle inflammatory status. Although fecal calprotectin has proved to be more sensitive as compared to CRP in predicting disease activity on endoscopy [19], it is less reliable in limited ileal disease [23, 24]. The composite use of both biomarkers has been shown to be more specific in detecting the inflammatory status in CD patients [25].

Several MRE severity indices have been proposed and evaluated from which the MaRIA score was found to have the best overall operational characteristics regarding the detection of active disease and in assessing the severity of disease [12]. This score was correlated to CD endoscopic index of severity and found wall thickness, presence of bowel wall edema, ulcer, and relative contrast enhancement as independent predictors of inflammation activity.

There are conflicting data regarding the correlation of the MaRIA score and inflammatory biomarkers. For instance, Cerrillo et al. [26] found significant correlation between MaRIA index and fecal calprotectin levels with AUC in ROC analysis of 0.914 and a cutoff value of 166.5 mcg/g yielded a 90% sensitivity and 74% specificity for the diagnosis of intestinal inflammation. On the other hand, Abej et al. [14] have shown that fecal calprotectin cutoff level of 250 mcg/g is not correlated with the MaRIA score.

In this study the addition of ADC and MIV to the MaRIA score has increased significantly the accuracy in discriminating active from nonactive disease.

DWI has been correlated with CD inflamed segments and with clinical scores. Stanescu-Siegmund et al. [27] demonstrated in 131 patients that areas of inflammation had significantly lower ADC values compared to normal bowel (*p* < 0.001) with threshold of 1.56 × 10^−3^ mm^2^/s having a sensitivity of 97.4% and specificity of 99.2% distinguishing normal and abnormal segments. Kim et al. [28] have shown that DWI increases the sensitivity in the detection of mild inflamed bowel segments with no obvious added value compared to conventional MR sequences in the detection of segments with ulcers. However, the addition of DWI to abnormal segments as seen on conventional MR sequences was correlated with higher endoscopy score (21 ± 10.1 versus 12.6 ± 8.4; *p* = 0.021) and the AUC in ROC analysis distinguishing segments with and without ulcers was significantly higher if DWI was added to conventional MR sequences (0.72 versus 0.661; *p* = 0.029). The specificity of DWI in detecting active disease was lower compared to conventional MR mainly due to false positive cases in the colorectal area.

The use of metabolic inflammatory volume in CD patients has been investigated in few studies. Jacene et al. [29] have shown on PET/CT that the product of metabolically active volume (corrected to background) and SUVmean is correlated with Crohn's disease endoscopy index of severity (CDEIS). Russo et al. [30] demonstrated that SUVmax corrected to body lean mass was superior to the total inflammatory volume in differentiating fibrotic from transmural inflammatory stenosis.

Cerrillo et al. [26] have shown that SUV-related metrics on 18F-FDG PET/CT correlated with CRP and recommended its use to monitor longitudinal changes of inflammation.

We found that the use of composite values of fecal calprotectin and CRP in combination to define active versus nonactive CD results in AUC 0.92 (83% sensitivity and 100% specificity using a cutoff value of 447) if MIV and ADC were added to the MaRIA score. This high association of 18F-FDG PET/MRE metrics with inflammatory biomarkers opens new opportunities for monitoring CD patients as both studies are noninvasive, quantifiable, and complementary.

Our study has several limitations: first, the number of patients is too small. Second, given the lack of standardization in DWI sequences and MIV measurements and for PET/MRE in general, a reliability study for these variables should be performed. Third, the low availability and high cost of PET/MR limit its use only to few academic centers.

In conclusion, in this pilot study, the addition of ADC and MIV to the MaRIA score increases the accuracy for discrimination of disease activity in patients with CD. Further larger studies are needed to validate these results and to evaluate if these variables can be used to monitor disease activity in patients with CD.
